# Oxygen-limited cellobiose fermentation and the characterization of the cellobiase of an industrial *Dekkera/Brettanomyces bruxellensis* strain

**DOI:** 10.1186/2193-1801-3-38

**Published:** 2014-01-20

**Authors:** Alexandre Libanio Silva Reis, Raquel de Fátima Rodrigues de Souza, Rochane Regina Neves Baptista Torres, Fernanda Cristina Bezerra Leite, Patrícia Maria Guedes Paiva, Esteban Espinosa Vidal, Marcos Antonio de Morais

**Affiliations:** Bioprocessing Laboratory, CETENE, 50740-540 Recife, PE, Brazil; Interdepartmental Research Group on Metabolic Engineering, Federal University of Pernambuco, 50670-901 Recife, PE, Brazil; Department of Genetics, Federal University of Pernambuco, 50670-901 Recife, PE, Brazil; Department of Biochemistry, Federal University of Pernambuco, 50670-901 Recife, PE, Brazil; Centro de Tecnologias Estratégicas do Nordeste - CETENE, Av. Prof. Luiz Freire, 01, Cidade Universitária, 50740-540 Recife, PE, Brasil

**Keywords:** BGL gene, β-glucosidase, Hydrolyzed bagasse, Lignocellulose

## Abstract

The discovery of a novel yeast with a natural capacity to produce ethanol from lignocellulosic substrates (second-generation ethanol) is of great significance for bioethanol technology. While there are some yeast strains capable of assimilating cellobiose in aerobic laboratory conditions, the predominant sugar in the treatment of lignocellulosic material, little is known about this ability in real industrial conditions. Fermentations designed to simulate industrial conditions were conducted in synthetic medium with glucose, sucrose, cellobiose and hydrolyzed pre-treated cane bagasse as a different carbon source, with the aim of further characterizing the fermentation capacity of a promising *Dekkera bruxellensis* yeast strain, isolated from the bioethanol process in Brazil. As a result, it was found (for the first time in oxygen-limiting conditions) that the strain *Dekkera bruxellensis* GDB 248 could produce ethanol from cellobiose. Moreover, it was corroborated that the cellobiase activity characterizes the enzyme candidate in semi-purified extracts (β-glucosidase). In addition, it was demonstrated that GDB 248 strain had the capacity to produce a higher acetic acid concentration than ethanol and glycerol, which confirms the absence of the Custer effect with this strain in oxygen-limiting conditions. Moreover, it is also being suggested that *D. bruxellensis* could benefit *Saccharomyces cerevisiae* and outcompete it in the industrial environment. In this way, it was confirmed that *D. bruxellensis* GDB 248 has the potential to produce ethanol from cellobiose, and is a promising strain for the fermentation of lignocellulosic substrates.

## Introduction

Recently, it has been demonstrated that the yeast *Dekkera bruxellensis* has the potential to ferment sucrose from sugarcane juice (Pereira et al. 2012; Leite et al. 2013). As well as being suitable for industrial production (de Souza Liberal et al. 2007), this yeast is a microorganism that can be used for ethanol fuel production. In addition, another useful characteristic is the ability to assimilate cellobiose, a disaccharide produced by the incomplete hydrolysis of cellulose. This capacity is of great importance in ethanol production from bagasse hydrolysates, where the waste material resources that are used, are inaccessible to the fermenting yeast *Saccharomyces cerevisiae* (Blomqvist et al. 2011). Cellobiose fermentation has been shown in *D. bruxellensis*, however, this feature is not present in all strains of this species (Blondin et al. 1982; Spindler et al. 1992; Blomqvist et al. 2010; Galafassi et al. 2011).

Two further well-documented features of *D. bruxellensis* are its ability to produce acetic acid from glucose, although only under aerobiosis conditions (Leite et al. 2013), and the Custer effect, the temporary inhibition of fermentation under anaerobic conditions (Wijsman et al. 1984; Scheffers 1979). In previous studies it has been suggested that *D. bruxellensis* is not able to produce glycerol (Gerós et al. 2000; Wijsman et al. 1984). However, we and others demonstrated that small amounts of glycerol are produced by this yeast (Pereira et al. 2012; Leite et al. 2013). In general, yeasts produce glycerol to redress the imbalance in redox potential, in anaerobic or oxygen-limiting growth conditions (van Dijken and Scheffers 1986).

In a previous study, we showed that *D. bruxellensis* strain GDB 248 is able to assimilate cellobiose when oxygen is supplied by flask agitation (Leite et al. 2013). In a continuation of this work, we are now showing that this *D. bruxellensis* strain GDB 248 can also ferment cellobiose under oxygen-limiting conditions and show the identification and partial characterization of the cellobiase activity (β-glucosidase, EC 3.2.1.21) and its encoding gene. There is also a discussion of the advantages and constraints of the biotechnological use of this yeast for second-generation ethanol production.

## Methods

### Yeast strain

*Dekkera bruxellensis* strain GDB 248 which is used in this study, is a wild strain isolated from the bioethanol industrial process (de Souza Liberal et al. 2005). Colonies of the strain were maintained by successive pitching in YPD agar plates.

### Bagasse hydrolysis

Steam-exploded sugarcane bagasse was suspended in 100 mM Tris-Acetate pH 4.5 buffer to 20 g/L and treated with Fibrenzyme™ LWT commercial preparation (Dyadic International Inc., Jupiter, USA), with 40 FPU/g of enzyme preparation for each 2% w/v of bagasse, at 50°C for 72 h with gentle agitation. The hydrolysate was centrifuged at 1,200 × g for 5 minutes and the liquid part was used for fermentation assays. The total sugar composition was evaluated by HPLC (as described below).

### Cultivations assays

Cells were pre-grown in liquid YPD at 30°C and 150 rpm for 24 h. Then, the yeasts were centrifuged at 4,500 × *g* for 10 min, and used for inoculation in the next stage. Industrial-like fermentations were performed with 10% (w/v) yeast biomass, in synthetic complete YNB medium (1.7 g/L) containing sucrose (25 g/L) (SMsuc), cellobiose (SMcello) (20.5 g/L) or a mixture of cellobiose and glucose (SMcello/glu) (10 g/L each), as well as in hydrolysed sugar cane bagasse (SMbag) (4.45 g/L cellobiose, 9.43 g/L glucose, 8.15 g/L xylose) at 30°C for different periods. Gentle agitation at 120 rpm was carried out to avoid cell sedimentation.

### Protein extract and cellobiase (β-glucosidase) activity

Yeast cells were cultivated in complete synthetic medium (1.7 g/L YNB) containing cellobiose at 1 g/L until 0.6 A_600nm_ and diluted 1/1000 to 250 mL fresh synthetic medium containing glucose, cellobiose or sucrose at 1.0 g/L. The flasks were incubated for 24 h at 33°C and 130 rpm in an orbital shaker. Afterwards, the cells were collected by centrifugation, re-suspended in 250 mL corresponding medium and cultivated (as described above). This process was repeated four times and after the last cycle, the final cell density was determined (Leite et al. 2013). All the cells were collected by centrifugation at 3,800 × *g* and 4°C for 30 min. The cell pellet was re-suspended in two volumes of 10 mM Acetate buffer pH 4 containing 1 mM β-mercaptoethanol and lysed by maceration in liquid nitrogen. The lysates were centrifuged at 21,000 × *g* for 15 minutes at 4°C and the supernatant was recovered. Protein concentration was determined by the Comassie® Blue method. The enzyme reaction was performed by mixing 100 μg of protein from a cell-free extract and sugar solution diluted in 100 mM sodium citrate buffer pH 4.8 for a final volume of one mL. The reaction was incubated for 10 min at 37°C and stopped by transferring the tubes to an ice bath. The release of glucose was measured with the aid of a glucose oxidase kit (LabLabor, Brazil). The specific activity was recorded as μmol of glucose released per minute from the amount of protein in one gram of yeast cells. When testing the presence of extracellular enzymes, supernatants of the cultures for sucrose or cellobiose were used for enzyme reactions.

### Cellobiase (β-glucosidase) purification

To obtain the protein extracts, yeast cells were grown in complete synthetic medium with cellobiose as a carbon source, lysed and subjected to fractioning in ammonium sulfate from 0% to 60% saturation. The precipitate was re-suspended in 100 mM sodium citrate buffer pH 5 and then dialyzed against deionized water for desalting protein fractions (Bollag et al. 1996). The protein fractions were tested for β-glucosidase activity using the chromogenic substrate p-nitrophenyl-β-D-glucopyranoside (pNPG). The fractions that showed enzyme activity were pooled (fraction EF1) and subjected to molecular exclusion chromatography in Sephadex® G75 (26 mm diameter, 10 cm height columns equilibrated with 100 mM citrate-phosphate buffer pH 5 at 6 mL/h). The fractions eluted containing β-glucosidase activity were pooled and subjected to ion exchange chromatography in CM-cellulose (15 mm diameter, 10 cm height columns equilibrated with 10 mM citrate-phosphate buffer pH 3.8 at 10 mL/h). Proteins linked to the matrix were eluted with 0.5 M NaCl solution at 10 mL/h flux and the fractions containing β-glucosidase activity were again pooled (fraction EF2). The purity of the proteins was checked by standard SDS-PAGE electrophoresis in 12% acrylamide gel. Isoelectrofocusing was performed to determine the isoelectric point of the protein. Immobilized pH Gradient (IPG) strips with pH ranging from 3 to 10 were equilibrated for 30 min with solution containing 6.5 mM DTT and 134 mM IAA and the protein was submitted to an electrophoretic run at 200 V (2 mA) for two hours followed by 3,500 V (2 mA) for 1.25 h. The strips were used for a second dimension run in 12% acrylamide gel and revealed by Comassie® Blue staining.

### Enzyme kinetic assays

Substrate specificity for disaccharides was determined (as described above). The kinetic profile of the EF2 fraction was evaluated using pNPG as substrate. Standard reactions used a volume of enzyme fractions containing 100 μg protein, an equal volume of 10 mM pNPG solution and 100 mM sodium citrate buffer pH 4.8 to one ml final volume. The reaction was incubated at 37°C for 10 minutes and stopped by adding 100 μL of 1 M sodium bicarbonate solution and the yellow color of pNP release was quantified at 410 nm. A standard curve was prepared with pNP to correlate the absorbance with the amount of the product released and the specific activity was calculated as the amount of enzyme that released one μmol pNP per minute per milligram of protein in the sample. Optimum pH was evaluated by using citrate-phosphate buffer adjusted for different pHs and the reactions were incubated at 30°C for 10 minutes. When testing the optimum temperature of cellobiase activity, the pH was adjusted to 4.0 and the reactions were incubated at different temperatures for 10 minutes. Thermal stability of the enzyme was analyzed by incubation with EF2 fraction for 10 minutes at temperatures ranging from 20°C to 60°C. Afterwards, the enzyme preparation was left at room temperature (ca. 25°C) for 10 min and then used for enzyme activity using pNPG at optimum pH and temperature. The maximum conversion rate (*V*_*max*_) and affinity constant (*K*_*M*_) were calculated from Lineweaver-Burk plot by varying pNPG concentration in the reactions, and were used to calculate the catalytic constant (Kcat) of the partially purified enzyme. Inhibitory activity was measured by adding disaccharides or pNPGal at 10 mM in reactions containing pNPG and expressed as the percentage of pNPG cleavage. All these measurements were performed at the optimum pH and temperature.

### Analysis of sugars and the main metabolic products

The concentration of ethanol, acetate, sucrose, glucose and cellobiose in the fermentation samples was determined by HPLC which comprises an Agilent 120039 system, an automatic injector, an infrared and UV detector and an AMINEX HPX-87H cation-exchange column (Bio-Rad, USA), preceded by a micro-Guard pre-column (Bio-Rad). The mobile phase used was sulfuric acid 5 mM at a flow rate of 0.6 ml/min. The oven temperature was 70°C. The sample injected was 20 μL. The compounds were identified by their relative retention times and quantified by direct comparison with a serial dilution and standard curve. The value represents the average of at least two biological replicates.

### Gene identification and *in silico* analysis

Searches through the keywords hydrolase, amylase, glucosidase and amyloglucosidase were performed in the *D. bruxellensis* Genomic Database (http://www.lge.ibi.unicamp.br/dekkera/) and the nucleotide sequences of the retrieved contigs were used for BLASTx analysis at GenBank. The *D. bruxellensis* contig which has a greater similarity to β-glucosidase encoding genes, was recovered and the ORF determined by the ORF Finder tool at NCBI. The partial sequence of the β-glucosidase protein was used for BLASTp analysis in the *D. bruxellensis* genome database of the Joint Genome Initiative-JGI (http://genome.jgi.doe.gov/Dekbr1/Dekbr1.home.html) to recover the complete protein sequence. Phylogenetic analysis of the amino acid sequences encoded by β-glucosidase genes of *D. bruxellensis* and other fungi, was performed as previously reported (de Souza Liberal et al. 2012). A sequence of the bacterial *Thermotoga neapolitana* β -glucosidase was used as the out-group. Functional domains of the putative β -glucosidase of *D. bruxellensis* were identified by using the structural analysis tools available online at the European Bioinformatic Institute (http://www.ebi.ac.uk/) and SIB Bioinformatic Resource Portal (http://www.expasy.org/).

### Statistical analysis

The data were analysed with ASSISTAT Software (7.6 beta Version) (Silva and Azevedo 2006). A series of seven tests was conducted to analyse the normal distribution of the variables (P > 10). Data with a normal distribution were analysed with parametric tests. The differences between the principal metabolites and higher alcohols measured in the fermentation samples, were determined by carrying out an analysis of variance (ANOVA) in a completely randomized design (p < 0.05). The Tukey Test was applied at a level of 5% of probability (p < 0.05) to determine the significant difference between the variables.

## Results

### Production and purification of *D. bruxellensis* cellobiase (β-glucosidase, E.C. 3.2.1.21)

With the aim of determining the best conditions for the expression of cellobiose activity, GDB 248 yeast cells were cultivated in synthetic media containing glucose, sucrose, maltose and cellobiose to test the activities of cell-free extracts for the main gluco-hydrolases: invertase (EC 3.2.1.26, β-fructofuranosidase), cellobiase (β-glucosidase, E.C. 3.2.1.21) and maltase (α-glucosidase, EC 3.2.1.20). No hydrolytic activity was detected in the supernatant of the cell cultures. The results of the analysis showed that intracellular invertase activity was 1.6 and 3.3-fold higher when cultivated in glucose, whereas the intracellular cellobiase activity was 3.4 and 1.8-fold higher in the sucrose than under cellobiose conditions (Table 1). Intracellular maltase activity was not detected in any of the conditions tested. However, it was only in the cellobiose growth conditions that the cellobiase activity was found to be around 1.6- times higher than with the invertase activity. To confirm the cellobiase expression, the cell-free extract prepared from the cellobiose cultivation of yeast cells was fractionated by ammonium sulfate precipitation and the fractions with cellobiase activity were pooled and partially purified (Table 2).

**Table 1:** **Effect of different carbon source medium on enzyme activity in the crude cell-free extracts from** ***Dekkera bruxellensis*** **GDB 248**

Sugar in the medium	Substrate of enzyme reaction	Enzyme assayed	Specific activity (U/mg protein)^a^
Glucose	Sucrose	Invertase	0.998 ± 0.01
	Cellobiose	β-glucosidase	0.835 ± 0.02
	Maltose	α-glucosidase	0.000
Sucrose	Sucrose	Invertase	0.598 ± 0.01
	Cellobiose	β-glucosidase	0.024 ± 0.03
	Maltose	α-glucosidase	0.000
Cellobiose	Sucrose	Invertase	0.295 ± 0.01
	Cellobiose	β-glucosidase	0.460 ± 0.02
	Maltose	α-glucosidase	0.000

^a^One unit of enzyme activity (U) refers to the μmol of glucose released per minute at 30°C from the amount of protein in one gram of yeast cells.

**Table 2:** **Summary of partial purification of cellobiase from** ***Dekkera bruxellensis*** **GDB 248 grown on cellobiose medium**

Purification step^a^	Protein concentration (mg/mL)	Specific activity (μmol EqGlucose/min.mgProtein)^b^	Purification factor
Cell-free extract	0.95	0.104	1.0
EF1^c^	1.19	0.113	1.09
EF2^d^	0.11	0.848	8.16

^a^Five grams of yeast pellet was used for protein extraction and purification.

^b^β-Glucosidase activity was assay with 100 mM of p-nitrophenyl-β-D-glucopyranoside (pNPG) at 30°C.

^c^Active fraction collected from molecular exclusion chromatography.

^d^Active fraction collected from ion exchange chromatography.

### Kinetic parameters of *D. bruxellensis* cellobiase

**Figure 1:**
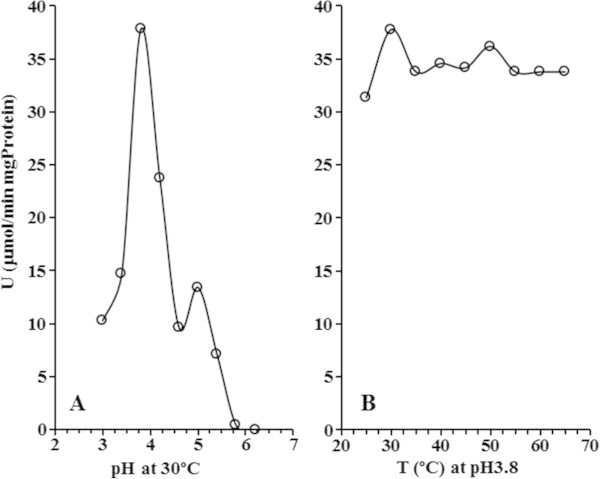
**Activity of β-glucosidase partly purified from** ***Dekkera bruxellensis*** **GDB 248 using pNPG as substrate at 37°C. (A)** Optimal pH and **(B)** temperature.

The purified cellobiase fraction candidate (referring to the EF2 fraction in Table 2) from *D. bruxellensis*, displayed a narrow range of pH, with its optimum activity in 100 mM sodium nitrate buffer at pH 3.8 when cellobiose was used as substrate (Figure 1A). On the other hand, EF2 showed a wider range of optimum temperatures (Figure 1B). Under these conditions (100 mM sodium citrate pH 3.8 and 30°C), the cellobiase from *D. bruxellensis* showed a broad spectrum of activity for disaccharides (Table 3). However, when the chromogenic synthetic disaccharide pNP-β-D-glucopyranoside (pNPG) was used, the optimum conditions for enzyme activity were established as 30°C and 5 mM sodium acetate buffer pH 4.8 and from this time onwards, all the experiments were performed in this condition. No activity was detected for the synthetic disaccharide pNP-β-D-galactopyranoside (pNPGal) (Table 3). Competitive inhibition of pNPG hydrolysis was observed in the presence of the cellobiose, sucrose and even maltose disaccharides, while the presence of pNPGal did not prevent an interaction of pNPG with the active site of the enzyme (Table 3). The following kinetic parameters were determined using pNPG: *K*_M_ = 0.58 mM; *V*_max_ = 154 μmol/min.mgProtein; *K*_cat_ = 12.84 min^-1^.

**Table 3:** **Effect of disaccharides on the activity of the purified cellobiase from** ***Dekkera bruxellensis*** **GDB 248**

Substrate	Glucosyl link	Relative activity (%)	Inhibitory activity (%)^a^
Cellobiose	Glucose-β(1 → 4)β-Glucose	100.0	100.0
Maltose	Glucose-α(1 → 4)α-Glucose	27.7 ± 0,01	94.9
Sucrose	Glucose-α(1 → 2)β-Fructose	90.0 ± 0,03	95.2
pNPG	Glucose-β(1 → 4)β-phenyl	100.0	na^b^
pNPGAL	Galactose-β(1 → 4)β-phenyl	0.0	0.0

^a^Dissacharides were added to reactions with the chromogenic substrate pNPG.

^b^not applicable.

### *In silico* analysis of *BGL* gene

The nucleotide sequence of the cellobiose encoded *Dekkera/Brettanomyces bruxellensis BGL* gene was identified from the *Dekkera bruxellensis* Genomic Database. The protein structure analysis predicted three major domains, similar to the *Kluyveromyces marxianus* enzyme. The N-terminus showed a glucosyl-hydrolase family 3 motif followed by the PA14 β-barrel, which is involved in carbohydrate binding in *K. marxianus* (Yoshida et al. 2010). At the C-terminus, there was a fibronectin type III-like domain that is also present in the structure of *K. marxianus* cellobiase (Yoshida et al. 2010). It is possible that this domain is involved in protein-protein interaction to form a homodimer structure of the enzyme. The results obtained *in silico* confirmed the identification of the putative *BGL* gene encoding cellobiose (β-glucosidase) of *D. bruxellensis.*

### Industrial-like fermentation

**Figure 2:**
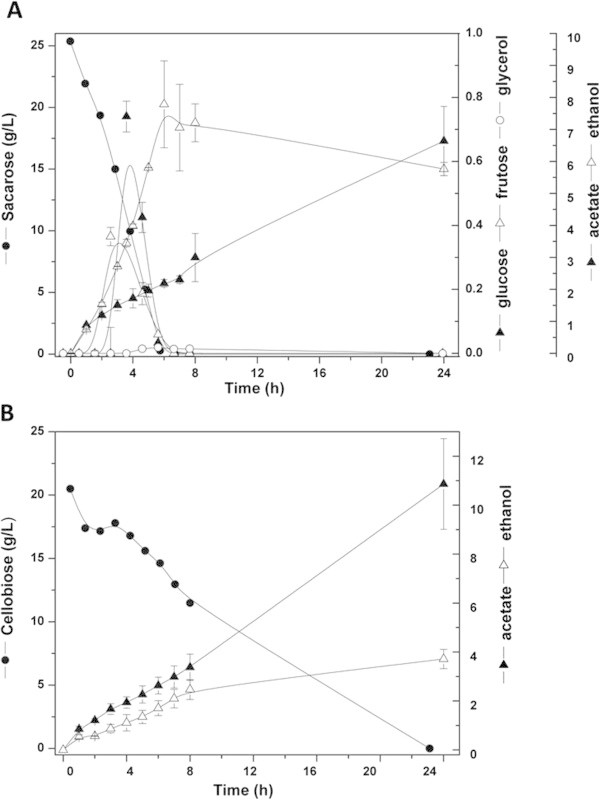
**Fermentative kinetics parameters of shake flask cultivation of** ***Dekkera bruxellensis*** **GDB 248.** Synthetic medium containing sucrose **(A)** or **(B)** cellobiose for 24 hours at 32°C in oxygen-limiting conditions.

A comparative fermentation assay was carried out using cellobiose as the carbon source to analyze the fermentation efficiency of GDB 248 strain with regard to the model for sucrose containing industrial fermentation. Figure 2 displays the kinetic profile of the GDB 248 strain in YNB synthetic medium with sucrose (SMsuc) (panel A) and cellobiose (SMcello) (panel B). It was calculated that the rate of sucrose consumption was 3.8-fold higher than cellobiose (Table 4). Although all the sucrose was consumed in the first 6 hours of fermentation, in the SMcello condition the cellobiose still remained above 50% at 8 h of fermentation (Figure 2B). This resulted in a 1.56-fold higher production of ethanol in the SMsuc condition. In contrast, the acetate production was 1.64-fold higher in cellobiose than in sucrose supplementation. No glycerol was detected in the course of the fermentation in either of the disaccharide carbon sources. At the same time, it was only in SMsuc conditions that increased concentrations of fructose and glucose were detected (at 1 g/L for four hours of fermentation declining to zero for 8 hours). No variation in the cell biomass was observed (data not shown) in any of the conditions applied.

**Table 4:** **Physiological parameters at the end of fermentation by** ***Dekkera bruxellensis*** **strain GDB 248**

Media^*^	Sugar	Sugar supplied (g/L)	Residual sugar (g/L)	q***S***(-g/L.h)	Glycerol (g/L)	Acetate (g/L)	Ethanol (g/L)	***P***_***max***_(g/L.h)	Fermentation efficiency (%)
SMSsuc	Sucrose	25.00	0.00	4.79	0.00	6.64(±1.08)^ab^	5.76(±0.21)^a^	0.24	42.85
SMScello	Cellobiose	20.50	0.00	1.26	0.00	10.87(±1.85)^a^	3.72(±0.39)^b^	0.15	33.71
SMScello/glu	Cellobiose	10.00	0.00	0.43	0.18(±0.04)^a^	4.73(±0.89)^b^	5.88(±0.49)^a^	0.25	56.06
	Glucose	10.00	0.00	2.89					
SMSbag	Cellobiose	4.45	3.41(±0.30)^a^	0.04	0.49 ±(0.04)^b^	5.38(±0.24)^b^	4.87(±0.40)^ab^	0.10	53.95
	Glucose	17.58	0.95(±0.06)^b^	0.18					

*Synthetic YNB medium containing sucrose (SMSsuc), cellobiose (SMScello), a mixture of cellobiose and glucose (SMScello/glu) or acid pre-treated enzyme-hydrolyzed sugarcane bagasse (SMSbag). Rates of sugar consumption (q*S*) and maximal ethanol production (*P*_*max*_) as well as the percentage of maximal theoretical yield were calculated. Average values (and standard deviation in parenthesis) were calculated from biological replicates with technical replicates for each sample. Similar superscript letters indicate no statistical difference according to ANOVA test (p < 0.05).

**Figure 3:**
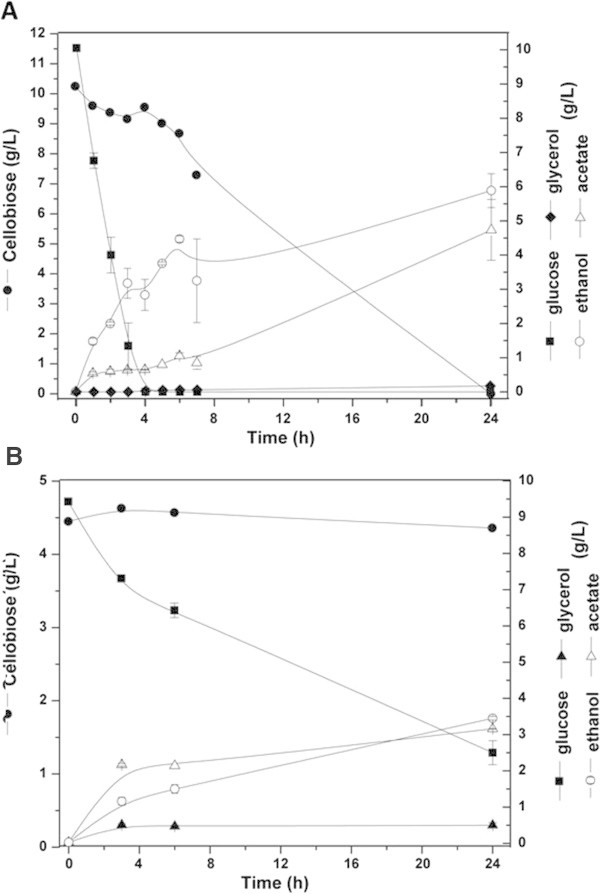
**Fermentative kinetics parameters of shake flask cultivation of** ***Dekkera bruxellensis*** **GDB 248 for 24 hours at 32°C in oxygen-limiting conditions. (A)** Synthetic medium containing a mixture of cellobiose and glucose. **(B)** Steam exploded sugarcane bagasse.

Following this, the fermentative parameters of assays which had a mixture of the same quantities of glucose and cellobiose, were compared so that a further analysis could be conducted of the sugar cane bagasse to assess the assimilation capacity of the GDB 248 strain. In Figure 3 the kinetic profile of GDB 248 strain is represented with a mixture of glucose and cellobiose (SMcello/glu) (A) and in sugarcane bagasse (B). The cellobiose and glucose curves showed that the GDB 248 strain displayed 17-fold and 10.5-fold higher consumed rate in SMcello/glu condition than in the SMbag (Table 1). Of all the conditions tested, glycerol production was found to be higher in the SMbag condition, with a change that was 2.8-fold more than in the SMcello/glu condition. With regard to acetate and ethanol, the production was similar for both conditions (p < 0.01), whereas the maximum productivity in SMcello/glu was 2.4-fold higher than in the SMbag condition, which, together with the SMsuc condition, was one of the highest production rates detected (0.24 g/L.h) (Table 4). Thus, it seemed that the fermentation of cellobiose was stimulated by the presence of glucose in the medium, while reducing acetate production and increasing fermentation efficiency (Table 4). This may be partially explained by the fact that glucose induced the cellobiase activity in the cell- free extract more than the other carbon sources tested in this study (Table 1).

## Discussion

The yeast *D. bruxellensis* strain has a reputation for causing spoilage in bioethanol and wine production and for being a dominant factor in these industrial processes (de Souza Liberal et al. 2005). However, its merit as a potential candidate for fermenting yeast in second-generation bioethanol production from lignocellulosic substrates has been reported (Blomqvist et al. 2011). In addition, the industrial strain has shown a relatively high performance in sugarcane juice fermentation (Pereira et al. 2012) and sugarcane molasses (manuscript in preparation) as well as being capable of using cellobiose as a carbon source (Leite et al. 2013).

Cellobiose assimilation and hydrolysis is of special concern when considering the production of ethanol from hydrolysed cellulosic material. In this study, the results demonstrated that under oxygen-limiting conditions the cells of GDB 248 strain are able to assimilate cellobiose with 64.5% relative efficiency compared with the assimilation of sucrose. The fermentation conditions employed in this work included high inoculum biomass (10% w/v), low agitation and high carbon concentration and the generation of high positive CO_2_ pressure. This ensured an almost anaerobic or oxygen-limited environment, which allowed us to evaluate the potential ability of the GDB 248 strain to produce ethanol in industrial-like conditions. This differed considerably from previous studies that reported cellobiose fermentation from other *D. bruxellensis* strains (Blomqvist et al. 2010; Galafassi et al. 2011). The GDB 248 strain preferred glucose as a carbon source to cellobiose, and this resulted in a higher rate of ethanol production and fermentation efficiency. This preference could be attributed either to the catabolic repression exerted by the glucose or to its limited capacity for cellobiose assimilation, *i.e.* a deficiency in The transporter.

The partial purification of a protein fraction was carried out to confirm the cellobiase activity and this resulted in the characterization of the β-glucosidase activity. The optimum conditions for enzyme activity (30°C and pH 4.8) and its kinetics profile resemble those outlined by *Kluyveromyces marxianus* (Yoshida et al. 2010). Moreover, the results obtained *in silico* identified the *BGL* gene encoding for β-glucosidase in the genome of *D. bruxellensis*. The theoretical protein contained 840 amino acids with a predicted molecular weight of 93 KDa and had a glucosyl-hydrolase family 3 motif at the N-terminus followed by the PA14 β-barrel, which was thought to be involved in carbohydrate binding in the *K. marxianus* enzyme (Yoshida et al. 2010). At the C-terminus there was a fibronectin type III-like domain that is also present in the structure of *K. marxianus* cellobiase (Yoshida et al. 2010), the function of which is still unknown. This domain might be involved in protein-protein interaction to form a homodimer structure of the enzyme. Neither the transmembrane nor the signal peptide domain nor the glycosylation sites were identified, which corroborates the experimental results which show the intracellular location of the enzyme.

Ethanol and acetate were produced under all the oxygen-limiting industrial conditions that applied here. Furthermore, only a very low production of glycerol was observed, as previous reported (Pereira et al. 2012; Leite et al. 2013). It is well known that in oxygen-limiting conditions in *D. bruxellensis*, *either* an inhibition of fermentation, or a Custer effect occurs. In addition, it was found that no acetic acid was produced while the glucose was being consumed at a high rate (Figure 3A). When cellobiose is consumed at a low rate, the production of acetate is accelerated (Figure 3A). These suggest there was glucose inhibition of aldehyde dehydrogenase, which is the enzyme responsible for acetate production (Blomqvist et al. 2011). In this study The results obtained demonstrate the capacity of *D. bruxellensis* to produce acetic acid even in oxygen-limiting conditions and points to the importance of the Custer effect in the final ethanol yield. Similarly, in the study by Blomqvist et al. (2010) noticeably more acetic acid was produced when *D. bruxellensis* was grown on cellobiose as a sole carbon source, than on glucose under the same conditions. Another interesting feature is that *Dekkera/Brettanomyces* yeast is sensitive to acetic acid concentrations above 2 g/L (Yahara et al. 2007), while GDB 248 strains proved to be very resistant to acetic acid - in the range of 5.5-fold higher than other strains. This acetate tolerance has recently been noted (Pereira et al. 2012). It seems that there are wide variations in the genome of different *Dekkera/Brettanomyces* strains, and this leads to a great variety of phenotypes.

## Conclusions

The results given here may help to explain why *Dekkera/Brettanomyces* yeasts can outcompete *S. cerevisiae* in industrial environments, while isolated cultures of *D. bruxellensis* in the same condition have low fermentation efficiency. It is possible that this behaviour can be attributed to the fact that both cellobiase and invertase are intracellular in *D. bruxellensis*. However, in the industrial oxygen-limiting conditions in which sucrose is the principal carbon source, the extracellular invertase activity from *S. cerevisiae* can be exploited by *Dekkera/Brettanomyces* yeasts.

It has been recently stated that there is an increasing number of yeasts capable of hydrolyzing cellobiose for the production of ethanol, for example *Candida queiroziae*, *Clavispora* sp. and *Spathaspora passalidarum* (Long et al. 2012; Lewis Liu et al. 2012; Santos et al. 2011), and *D. bruxellensis* (Blomqvist et al. 2010; Galafassi et al. 2011; Leite et al. 2013). However, only the *Dekkera/Brettanomyces* species has proved to be able to settle and survive in industrial environments (Passoth et al. 2007; de Souza Liberal et al. 2007; Pereira et al. 2012). Thus, the great challenge for its use as a fermenting yeast is how to address the question of the low conversion rates, when faced with simulated industrial conditions. Studies are being undertaken in our laboratory to identify the main metabolic bottlenecks that characterize this feature and to evaluate the capacity of the species to convert hydrolysates of sugarcane and sweet sorghum bagasse into ethanol and achieve high industrial yields.
